# Correlation of the middle cerebral artery atherosclerotic plaque characteristics with ischemic stroke recurrence: a vessel wall magnetic resonance imaging study

**DOI:** 10.18632/aging.204950

**Published:** 2023-08-10

**Authors:** Fangbing Li, Yilin Wang, Ying Du, Tianxiang Hu, Yejun Wu

**Affiliations:** 1 Department of Radiology, Fourth Affiliated Hospital of China Medical University, Shenyang, China

**Keywords:** vessel wall magnetic resonance imaging, atherosclerotic plaques, middle cerebral artery, ischemic stroke recurrence, plaque

## Abstract

This study aims to analyze the imaging features of atherosclerotic plaques in the middle cerebral artery (MCA) of patients with recurrent ischemic stroke using vessel wall magnetic resonance imaging (VWMRI) and investigate the correlation between these imaging features and the recurrence of ischemic stroke. Consecutive patients with ischemic stroke caused by atherosclerotic stenosis of the MCA were collected. The patients were divided into recurrent and non-recurrent ischemic stroke groups. We obtained VWMRI images of MCA plaques using 3.0T MRI by black-blood sequences, and the differences in VWMRI characteristics and clinical information between the two groups were compared. A binary Logistic regression model was used to analyze the VWMRI characteristics and clinical information related to ischemic stroke recurrence. 179 patients were collected from August 2018 to May 2020, and 81 patients were included in the study. The recurrent ischemic stroke group patients had a higher stenosis rate (0.69 vs 0.64). Meanwhile, the rate of centripetal wall thickening was significantly higher in patients with recurrent ischemic stroke (33.3% vs 11.7%). Binary Logistic regression analysis showed that sex (P=0.036, OR:2.983, CI:1.075-8.279), stenosis rate (P=0.038, OR:148.565, CI:1.331-16583.631), and vessel wall thickening pattern (P=0.012, OR:0.171, CI:0.043-0.678) were related to ischemic stroke recurrence. The patients with ischemic stroke caused by atherosclerotic stenosis of MCA, female patients, and those with concentric wall thickening and a high degree of stenosis have a higher risk of recurrence. Our results suggest that VWMRI is a valuable tool for predicting the risk of ischemic stroke recurrence in patients with MCA plaques.

## INTRODUCTION

In recent years, the incidence of ischemic stroke has been increasing worldwide, and the occurrence and recurrence of ischemic stroke have become a major cause of death and disability in China [1]. Many risk factors contribute to the occurrence and recurrence of ischemic stroke, such as hypertension, diabetes mellitus, atherosclerotic lesions, etc. [2]. Intracranial arterial stenosis due to atherosclerosis is one of the major causes of ischemic stroke and is the most common cause in Asian populations [3].

Studies have shown that the risk of recurrence within one year after ischemic stroke is 3.54-24.5% and up to 53% within five years [4, 5]. Compared with the first episode of ischemic stroke, patients with recurrent ischemic stroke tend to have worse neurological status, poorer prognosis, and higher disability rates. Therefore, preventing ischemic stroke recurrence is an important clinical issue. It is necessary to investigate the risk factors associated with recurrent ischemic stroke, including both clinical and imaging characteristics of stroke patients. Studies have shown that the severity of intracranial arterial stenosis is an independent risk factor for stroke in the area of arterial stenosis [6, 7]. In patients with stenosis degree ≥70%, the risk of recurrent stroke in the area of arterial stenosis is as high as 23% in the first year [8]. However, it is not comprehensive to evaluate only the degree of stenosis. One study showed that half of the patients with acute ischemic stroke had less than 50% intracranial artery stenosis [9].

Compared to conventional imaging methods for evaluating arterial stenosis (e.g., MRA, CTA, etc.), vessel wall magnetic resonance imaging (VWMRI) is a reliable examination method that enables the *in-vivo* analysis of the intracranial vessel wall and lumen. Using the black blood technique, VWMRI can evaluate the stenosis, the vessel wall, and the characteristics of the atheromatous plaque causing arterial stenosis [10].

The middle cerebral artery (MCA) is a major branch of the intracranial segment of the carotid artery and is most susceptible to atherosclerotic lesions that can cause ischemic stroke. Therefore, we analyzed the imaging characteristics of MCA plaques in patients with ischemic stroke recurrence by VWMRI. We hypothesized a correlation between the imaging characteristics of the MCA atherosclerotic plaques and ischemic stroke recurrence. This study aimed to identify VWMRI imaging features that can potentially predict the risk of ischemic stroke recurrence and thus provide a useful reference for prevention and treatment strategies in these cases. The clinical significance of this study lies in the potential for our findings to further demonstrate VWMRI as a valuable tool in predicting the risk of recurrent ischemic stroke among patients with MCA plaques.

## RESULTS

### Patients clinical information

From August 2018 to May 2020, 179 patients were recruited for this study based on the inclusion criteria. However, 98 individuals were excluded from the study (23 with substandard image quality, 36 with moderate or higher carotid stenosis, 24 with atrial fibrillation, 12 with moyamoya disease, and 3 with MCA dissection). Finally, 81 patients were included in the study (30 with ischemic stroke recurrence and 51 with ischemic stroke non-recurrence).

Detailed clinical information of the patients is shown in Table 1. Only sex (p=0.014) was statistically significant in recurrent and non-recurrent ischemic stroke patients. All patients received antiplatelet drugs to aid treatment, and patients with diabetes, hypertension, and hyperlipidemia were administered medication commensurate with their respective health conditions. Among the patients with recurrent ischemic stroke, two had poor blood pressure control, three had poor serum lipoprotein control, and five had poor control of blood glucose levels. Conversely, of the patients without recurrent ischemic stroke, seven had poor blood pressure control, four had poor control of blood glucose levels, and twelve had poor serum lipoprotein control. The two groups had no significant statistical significance in drug treatment (P≤0.05).

**Table 1:** Patients clinical information.

	**Ischemic stroke recurrence (n=30)**	**Ischemic stroke non-recurrence (n=51)**	***P***
Sex			0.014
Male	11(36.7%)	33(64.7%)	
Female	19(63.3%)	18(35.3%)	
Age	64.21±13.12	59.37±14.21	0.110
Hypertension	23(76.7%)	35(68.6%)	0.266
Diabetes	14(46.7%)	18(35.3%)	0.312
Dyslipidemia	16(53.3%)	40(78.4%)	0.336
Hyperhomocysteinemia	8(26.7%)	23(45.1%)	0.458
Smoking (ever)	10(33.3%)	26(51.0%)	0.123
Alcoholism (ever)	6(20.0%)	11(21.6%)	0.867
NIHSS	1(0-3)	1(0-4)	0.547

NIHSS, national institutes of health stroke scale.

### Comparisons of imaging features of plaques in patients

The intra-observer (ICC=0.971, P<0.05) and inter-observer (ICC=0.912, P<0.05) consistency of all imaging features evaluations was good. Among the imaging features of the plaque, there were statistically significant differences in the stenosis rate (P=0.034) and the pattern of wall thickening (P=0.003). The patients in the recurrent ischemic stroke group had a higher stenosis rate (0.69 (0.65-0.77)) than those in the non-recurrent ischemic stroke group (0.64 (0.54-0.63)). Meanwhile, the rate of centripetal wall thickening was significantly higher in patients with recurrent ischemic stroke (n=10; 33.3%) than those without recurrence (n=6; 11.7%). However, normalized wall index (NWI), plaque location, plaque surface and fibrous cap characteristics, and whether the plaque involved perforating arteries showed no statistically significant differences (Table 2).

**Table 2:** Patients imaging characteristics.

		**Ischemic stroke recurrence (n=30)**	**Ischemic stroke non-recurrence (n=51)**	**P**
Stenosis rate		0.69(0.65-0.77)	0.64(0.54-0.63)	0.034
NWI		0.76(0.71-0.84)	0.72(0.55-0.85)	0.148
The pattern of wall thickening				0.003
	Eccentric thickening	20(66.7%)	45(88.3%)	
	Centripetal thickening	10(33.3%)	6(11.7%)	
Plaque location *				0.113
	Superior	4(28.6%)	11(21.6%)	
	Inferior	7(23.3%)	14(27.4%)	
	Ventral	9(64.3%)	18(35.3%)	
	Dorsal	0(0.0%)	2(3.9%)	
RR		0.92(0.76-1.00)	0.96(0.91-1.04)	0.066
Plaque surface characteristics	Smooth	4(13.3%)	24(47.1%)	0.747
	Irregular	17(56.7%)	27(52.9%)	
Fibrous cap characteristics	Uniform	5(16.7%)	13(25.5%)	0.493
	Non-uniform	25(83.3%)	38(74.5%)	
Involvement of perforating arteries	Yes	5(16.7%)	5(9.8%)	0.368
	No	25(83.3)	46(90.2%)	

NWI, normalized wall index, RR, remodeling ratio; *: Plaque location refers to the distribution of plaque in patients with eccentric wall thickening.

### Correlation analysis of ischemic stroke recurrence

**Figure 1:**
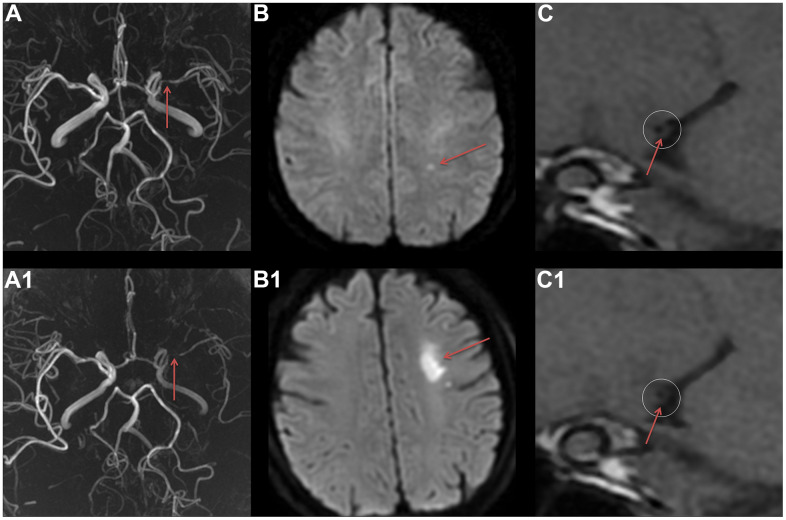
**The VWMRI of MCA plaque characteristic in a patient with ischemic stroke recurrence.** A female patient, 56 years old, experienced her first ischemic stroke in August 2019. MRA revealed severe stenosis of the M1 segment of the left MCA (**A**). At the same time, DWI displayed a punctate high signal in the left centrum semiovale (**B**), and VWMRI revealed centripetal thickening of the MCA and severe lumen stenosis (**C**). In November 2019, the patient experienced a recurrent ischemic stroke. MRA displayed an aggravated stenosis of the M1 segment of the left MCA (**A1**), and DWI showed a new patchy high signal in the left centrum semiovale (**B1**). Furthermore, VWMRI revealed an aggravated centripetal thickening of the MCA and a worsening lumen stenosis (**C1**).

A correlation analysis was conducted on factors associated with recurrent ischemic stroke, and those factors where P<0.05, including sex, stenosis rate, and pattern of wall thickening, were included in the regression equation as covariates. The results showed that sex (P=0.036, OR:2.983, CI:1.075-8.279), stenosis rate (P=0.038, OR:148.565, CI:1.331-16583.631), and pattern of wall thickening (P=0.012, OR:0.171, CI:0.043-0.678) were significantly correlated with recurrence (Figure 1).

## DISCUSSION

This study investigated the association between risk factors and recurrent ischemic stroke caused by atheromatous stenosis in the MCA by comparing VWMRI imaging features and clinical information between patients with recurrent and non-recurrent ischemic stroke. Our analysis revealed statistically significant differences in sex, stenosis rate, and mode of wall thickening between the two groups of patients. Furthermore, using binary Logistic regression analysis, we found that female patients, concentric wall thickening, and high stenosis rate were associated with a higher risk of recurrent ischemic stroke in northern China. These findings highlight the importance of considering these risk factors in developing preventive measures for recurrent ischemic stroke in patients with atheromatous stenosis in the MCA.

Previous studies have broadly defined recurrent stroke as focal neurological function defects lasting more than 24 hours after the initial stroke without considering the time interval between the occurrences [11, 12]. In this study, we included patients who experienced recurrent stroke with focal neurological function defects lasting for over 24 hours (including TIA) within two years. One study has shown that the recurrence rate of stroke at 1, 2, 3, 5, and 10 years after the first ischemic stroke is 10.4%, 16.1%, 16.7%, 14.8%, and 12.9%, respectively [13]. The risk of recurrent stroke remains relatively stable between two to five years after the onset of initial ischemic stroke.

Our study in a population from northern China found that female patients with ischemic stroke have a higher recurrence rate than male patients, which is consistent with some previous studies on recurrent ischemic stroke [14, 15]. However, other studies have identified the male sex and black race as independent risk factors for recurrent stroke [16, 17]. Generally, the mechanisms and causes of sex differences in stroke recurrence are intricate. Based on the analysis of our findings, there may be several potential reasons to explain the sex difference in stroke recurrence. Firstly, the patients in our study were older, and the increased risk of stroke recurrence may be related to the loss of the neuroprotective effects of estrogen in postmenopausal women [18]. Furthermore, the clinical risk factors for stroke occurrence and recurrence include hypertension, smoking, alcohol abuse, diabetes, etc. Women are more affected by these risk factors than men [18]. Additionally, the elevated incidence of recurrent stroke among women than men may be partly due to women's longer life expectancy, for which age is a nonmodifiable risk factor [19]. Since our study only included patients from the same geographical area, further large-scale, multicenter studies involving diverse populations are necessary to explore the influence of sex on the recurrence risk of ischemic stroke in different regions and ethnic groups.

VWMRI is a highly effective non-invasive method for evaluating intracranial atherosclerotic plaques. VWMRI enables the evaluation of the vessel lumen and wall, making it an essential tool for comprehensively assessing these plaques. VWMRI can complement conventional imaging methods in evaluating intracranial arterial plaque, providing more comprehensive imaging information and key insights for treatment decisions regarding intracranial atherosclerotic lesions [20]. In general, the analysis of VWMRI encompasses the evaluation of plaque structure (including vessel wall thickening patterns caused by plaque, vessel wall remodeling, plaque surface morphology, and plaque location) and tissue composition (intraplaque hemorrhage, fibrous cap signal characteristics, and plaque enhancement), which usually combined with lumen analysis (lumen stenosis rate and plaque burden) [21]. Through plaque analysis in this study, we have identified a clinical relevance between the rate of luminal stenosis and concentric thickening of the vessel wall to recurrent ischemic stroke.

Our study found that patients with higher stenosis rates have a higher risk of recurrent stroke, which is consistent with previous research conclusions [9]. The degree of stenosis has always been a key concern for clinicians in diagnosing and treating ischemic stroke. Generally, more severe stenosis is associated with lower cerebral blood perfusion in the supply territory, resulting in an increased risk of ischemic stroke. The pattern of wall thickening is associated with ischemic stroke recurrence, and the risk of ischemic stroke recurrence was higher in this study with centripetal wall thickening. The circumferential distribution of plaques with centripetal wall thickening may lead to a greater incidence of involvement of small penetrating artery openings and a generally higher degree of stenosis. Some studies have suggested that patients with penetrating artery involvement have a higher risk of stroke recurrence [15, 22]. Therefore, both circumferential and superior wall plaques should be given adequate attention by clinicians.

Our study has some limitations. Firstly, previous studies have explored the correlation between plaque enhancement and recurrent stroke [16, 23]. However, we could not include plaque enhancement in our study due to the limited number of patients who underwent contrast-enhanced VWMRI. The main reasons include that enhanced VWMRI requires injection of contrast medium, and some patients with severe symptoms cannot tolerate the examination. At the same time, the examination time of enhanced VWMRI is prolonged, which leads to motion artifacts in some patients and affects the image quality. Secondly, our study is a single-center retrospective study with a limited sample size, and we could not perform a stratified analysis of the included patients based on clinical risk factors. Further large-scale multicenter trials are needed to investigate the risk factors for recurrent stroke.

## CONCLUSIONS

The patients with ischemic stroke caused by atherosclerotic stenosis of MCA in northern China, female patients, and those with concentric wall thickening and a high degree of stenosis have a higher risk of recurrence. Our results suggest that VWMRI is a valuable tool for predicting the risk of ischemic stroke recurrence in patients with MCA atherosclerotic plaques. The specific imaging features identified in this study can serve as a valuable reference for clinicians in preventing and treating ischemic stroke recurrence.

## MATERIALS AND METHODS

### Research subjects

This was a retrospective study that received approval from the Ethics Committee of our hospital (EC-2022-KS-022) with a waiver of patient informed consent. Recurrent ischemic stroke was defined as the reappearance of a focal neurological impairment lasting 24 hours or more within two years following the initial ischemic stroke. Based on this study's inclusion and exclusion criteria, participants with recurrent and non-recurrent ischemic stroke were consecutively collected from the Department of Neurology between August 2018 and May 2020. Clinical information was gathered from participants, including age, sex, hypertension history, diabetes, dyslipidemia, alcoholism, smoking history, hyperhomocysteinemia, National Institutes of Health Stroke Scale (NIHSS) score, and medication history.

In this study, the inclusion criteria consisted of participants who met the following conditions: 1. previous confirmation of recurrent or non-recurrent ischemic stroke by a neurologist; 2. imaging evidence that the MCA was responsible for the ischemic stroke; 3. confirmation of ischemic lesions in the ipsilateral MCA blood supply area via diffusion-weighted imaging (DWI); 4. clinical symptoms supporting the occurrence of ischemic stroke in the ipsilateral MCA blood supply area; and 5. patients underwent VWMRI and had a complete clinical history of the disease. Conversely, participants were excluded if they met the criteria: 1. congenital vascular malformation; 2. carotid artery stenosis exceeding 50%; 3. previous MCA angioplasty or stenting; 4.MCA stenosis resulting from a factor other than atherosclerotic lesions (e.g., moyamoya disease, arterial dissection, or vasculitis); 5. cardiogenic arterial embolism; or 6. insufficient imaging quality for diagnosis.

### MRI protocol

VWMRI was performed using a 3.0T MRI (GE MEDICAL SYSTEMS DISCOVERY MR750, GE Healthcare, USA) with an 8-channel head coil, and the scan parameters are shown in Table 3. The scanning scope included the diseased vessels of the MCA.

**Table 3:** Magnetic resonance imaging protocol.

**Sequences**	**TR (ms)**	**TE (ms)**	**Flip angle**	**Slice thickness (mm)**	**FOV (mm)**	**NEX**	**Locs per slab**	**Matrix**	**Acquisition time**
3D TOF-MRA	23	2.5	20	1.4	220	3	32	320×256	4 min 1 s
3D CUBE T1WI	1140	14	/	1	180	1	160	320×228	7 min 5 s
2D FSE T2WI	4000	42	125	2	130	4	16	256×224	6 min 8 s
DWI	3000	61.8	/	5	240	1	18	128×130	24s

3D, three dimensional; 2D, two dimensional; DWI, diffusion-weighted imaging; TR, repetition time; TE, echo time; FOV, field of view; NEX, number of excitations; Locs per slab, total number of locations (slices) generated from a slab; min, minute; s, second; CUBE, variable-flip-angle turbo-spin-echo.

### Definition of VWMRI plaque characteristics

**Figure 2:**
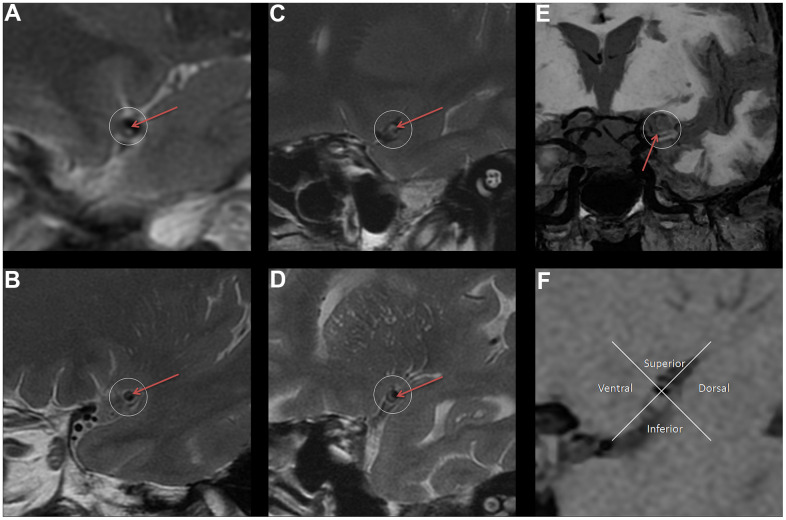
**The legend introduction of VWMRI plaque characteristics.** (**A**) Plaque surface is irregular (red arrow). (**B**) Plaque surface is smooth (red arrow). (**C**) Uneven thickness of fibrous cap (red arrow). (**D**) Uniform thickness of fibrous cap (red arrow). (**E**) Plaque involves the opening of the lenticulostriate arteries (red arrow). (**F**) Distribution of plaque (The plaque is mainly located in the inferior).

Stenosis rate (%) = [1-(stenosis lumen diameter/normal lumen diameter)] × 100%, stenosis lumen diameter is the lumen diameter at the most severe stenosis of the diseased vessel [24]. NWI = (vessel area - lumen area)/vessel area × 100%, the lumen area and vessel area at the narrowest vessel are usually measured to calculate the NWI [25]. The Remodeling ratio (RR) is the ratio of the diseased vascular area to the normal vascular area [25]. Eccentricity index = (maximum vessel wall thickness - minimum vessel wall thickness) / maximum vessel wall thickness; if the eccentricity index is ≥ 0.5, the vessel is defined as an eccentric thickening; otherwise, it is centripetal thickening [25]. Plaque location is defined as the distribution of plaques with eccentric wall thickening across the vessel and can be divided into four segments (ventral, dorsal, superior, and inferior) [26]. Plaque surface and plaque fibrous cap characteristics: according to the imaging characteristics of VWMRI images, the evaluation is usually divided into whether the plaque surface is smooth and whether the fiber cap thickness is uniform [27]. Finally, plaque characterization is also required to determine whether the plaque involves the opening of the lenticulostriate arteries on cross-sectional images (Figure 2).

### Image measurement and evaluation

The images were graded according to the display of the vessel wall, vessel lumen, and plaque (Level 1: poor image quality and undiagnosable. Level 2: Image quality is average and cannot meet all diagnostic requirements; Level 3: Image quality is good and meets diagnostic requirements). Image quality was graded by a radiologist. The image quality must reach level 3 before further image feature analysis can be carried out. All images were measured and evaluated separately and independently by two other radiologists according to evaluation criteria. Clinical information for all patients in the assessment was not visible. Measurements of continuous variables were statistically analyzed using the mean of measurements taken by two radiologists. Evaluation of grade variables If there was any disagreement in the evaluation results, a third radiologist was brought in to evaluate and reach a consensus through discussion. After four weeks, all images were re-assessed and re-measured by one of the radiologists using the same criteria.

All images were analyzed and measured using the Vessel Mass (Leiden University Medical Center, Leiden, the Netherlands) software. Stenosis rate, NWI, RR, plaque distribution, eccentricity index, and involvement of the lenticulostriate arteries were evaluated on 3D T1WI. Plaque surface and fibrous cap morphology were evaluated on 2D T2WI.

### Statistics

Statistical analyses in this study were carried out using IBM SPSS Statistics R26.0 software. Count data were presented as numerical values and percentages, whereas non-normally distributed measurements were expressed as median and percentile (P25%-P75%). Categorical variables were assessed using the chi-square test or continuous corrected chi-square test, rank variables by Wilcoxon signed rank sum test, and non-normally distributed measurements by the Mann-Whitney U test. The intra-group correlation coefficient (ICC) was utilized to evaluate the consistency of observers, where a value of ICC>0.80 indicated good consistency, 0.40≤ ICC ≤ 0.80 indicated fair consistency, and ICC<0.4 represented poor consistency. Factors yielding p<0.05 in the univariate analysis were used as covariates in multivariate analysis, and binary logistic regression was employed to determine the factors associated with stroke recurrence. A p-value <0.05 was considered to indicate statistical significance.
